# High and Low Dose Ketamine in Post-Traumatic Stress Disorder

**Published:** 2025-05-05

**Authors:** Joey Day, Devendra K. Agrawal

**Affiliations:** Department of Translational Research, College of Osteopathic Medicine of the Pacific, Western University of Health Sciences, Pomona, California 91766 USA

**Keywords:** Anesthesia, Glutamate, Ketamine, Plasticity, Post-traumatic stress disorder, N-methyl-d-aspartate, N-methyl-d-aspartate Receptor

## Abstract

Ketamine has unique properties making it a desirable drug to induce general anesthesia during surgery. However, it is typically reserved for children and adolescent patients due to its side effects in adults, including vivid dreams, hallucinations, and confusional states that may be pleasant or distressing. These symptoms may affect patients with post-traumatic stress disorder (PTSD). PTSD is a trauma-related psychological disorder that is mainly characterized by intrusive thoughts, hypervigilance, and re-experiencing of the trauma event. Most of current research focuses on the use of sub-anesthetic doses of ketamine as a treatment for PTSD. Limited information is known about high-dose ketamine use during general anesthesia and the impact this has on patients who suffer from PTSD. A literature review investigating the effects of anesthetic doses of ketamine on PTSD was conducted for this article. Findings suggest that ketamine has dose-related effects on the severity of PTSD. Specifically, low-dose ketamine has the potential as a therapeutic agent in the treatment of PTSD, while high-dose ketamine may cause worsening of PTSD symptoms. This could occur through the increase in psychomimetic symptoms, decrease in plasticity and metaplasticity, and modulation of fear memory systems experienced with anesthetic doses of ketamine. Currently, there are no published research articles directly measuring the effects of high-dose ketamine on PTSD. Further investigation is warranted to understand if anesthetic doses of ketamine worsen PTSD symptoms. This is important because it can help guide the management approach of an anesthesiologist to safely providing anesthesia to PTSD patients.

## Introduction

Ketamine was first synthesized in 1962 as a derivative of phencyclidine (PCP) to be used as a potent sedative and anesthetic [1]. Ketamine provides a unique type of anesthesia termed “dissociative anesthesia” due to its psychodysleptic features (i.e., hallucinations, dreams, delirium), cataleptic features, and feelings of detachment from reality [2].

In addition to its sedative effects, ketamine also provides analgesia and amnesia [3]. These properties make ketamine ideal for surgical anesthesia because it completes the so-called “triangle of anesthesia, “ which is constituted by anesthesia, analgesia, and amnesia. Usually, a careful balance of propofol, benzodiazepines, and opioids is required to complete the triangle of anesthesia. This means ketamine could be used as a single agent, avoiding the use of multiple drugs and their corresponding side effects.

Although these properties make ketamine a strong candidate for use during anesthesia, it is mostly reserved for children and adolescents due to its negative effects on adults. Namely, the emergence phenomenon, which occurs when emerging from anesthesia. It is characterized by vivid dreams, delirium, and hallucinations that may be enjoyable or distressing [4]. Also, ketamine can cause psychomimetic symptoms that resemble those seen in schizophrenia [5]. Ketamine transiently causes positive symptoms, such as hallucinations and delusions, and negative symptoms of schizophrenia in patients with or without clinically diagnosed schizophrenia. The positive symptoms are more prevalent [6]. The psychomimetic symptoms are often considered distinct from the dissociative symptoms which are better characterized as feeling detached from reality or “floating” [2].

Other adverse effects of the drug include an increase in blood pressure and heart rate, an increase in intracranial pressure, and respiratory depression. However, ketamine is considered relatively hemodynamically stable compared to other anesthetic agents [7]. Although respiratory depression is a documented side effect, it is rarely seen [8].

## Post Traumatic Stress Disorder: An Overview

Post traumatic stress disorder (PTSD) is a trauma-related psychopathy that most commonly occurs after exposure to a life-threatening situation involving an individual or a person close to them. Typical inciting traumas include military combat experiences and sexual assault [9]. The lifetime prevalence of PTSD has been estimated at 7.8% [10].

Symptomatology is grouped into four clusters: avoidance symptoms, re-experiencing symptoms, negative changes in cognition and mood, and alterations in arousal. Avoidance symptoms center around avoiding people, places, or objects that remind someone of the trauma. Re-experiencing symptoms can manifest as nightmares or recurrent intrusive thoughts of the event. Hypervigilance is classic and patients will appear restless, agitated, and distracted. Negative changes in mood and cognition include feelings of depression, dissociation, and guilt [11].

Not all people exposed to severe trauma develop PTSD, but the pathophysiology of PTSD remains largely unknown. Like most diseases, PTSD is a balance of genetic predisposition and environmental factors. A twin study reported the heritability of PTSD as 49% [12]. There is a positive correlation between the severity and frequency of traumatic events and the likelihood of developing PTSD [13]. Other risk factors include female gender, lower education level, and disadvantaged social status [11].

The preferred first-line treatment of PTSD is trauma-focused psychotherapy, while the first-line pharmacological treatment is selective serotonin reuptake inhibitors (SSRI) or selective serotonin-norepinephrine reuptake inhibitors (SNRI). Second-line pharmacological treatments include nefazodone, mirtazapine, tricyclic antidepressants, and monoamine oxidase inhibitors [14]. Prazosin can be used to treat nightmares in PTSD [15].

## Ketamine and Psychiatric Illness

This paper addresses the effect of high-dose ketamine in patients with PTSD. Ketamine may be administered in either subanesthetic or anesthetic doses. Subanesthetic doses are considered as 0.5 mg/kg IV or less. Anesthetic doses range from 1.0–2.0 mg/kg IV or greater but are better defined as the dose at which a patient is unconscious and unresponsive to verbal and tactile stimuli.

The use of low-dose ketamine is being investigated as a treatment for major depressive disorder (MDD) and PTSD. The FDA has approved Esketamine, a ketamine analog administered intranasally, for the treatment of MDD [16]. There is also vast evidence in both human and animal models suggesting a rapid reduction in symptoms of PTSD with carefully titrated low-dose ketamine [17,18]. Drugs with rapid onset are advantageous because the current first-line pharmacological agents (i.e., SSRI and SNRI) of MDD and PTSD take up to six weeks to achieve the desired therapeutic effects [19]. Although subanesthetic doses of ketamine are promising as a potential treatment of PTSD and MDD, the use of anesthetic doses does not exhibit the same effects.

The rapid reduction of MDD symptoms due to ketamine is found at low doses of ketamine but not at high doses. This was discovered using a well-established behavioral model of despair in rodents called the forced swim test (FST). The study found that low doses produced antidepressant activity while high doses did not [20]. Even though this study is investigating the anti-depressant effects of low- and high-dose ketamine, similar pathways are thought to be involved in ketamine’s effects on PTSD [21]. Therefore, it’s reasonable to suspect that high-dose ketamine would not have the same mitigating effects on PTSD symptoms as low-dose ketamine. This addresses the misconception that ketamine generally improves symptoms of PTSD and MDD. It should be clear that anesthetic doses of ketamine are most likely not effective in treating PTSD.

## Ketamine-NMDA Receptor Interaction

The primary mechanism of action of ketamine is the non-competitive antagonism of N-methyl-d-aspartate (NMDA) receptors in the central nervous system. However, it also acts on opioid, muscarinic, and various voltage-gated receptors [22]. Ketamine is typically a racemic mixture of (R)- and (S)-enantiomers in which (S)-ketamine has a greater affinity for the NMDAR and a longer half-life [23,24]. It is believed that ketamine binds to a site within the ion channel thereby directly blocking the channels exclusively when they are in the open state, and does so in a voltage-dependent manner [25]. This contributes to its non-specific inhibition of NMDAR.

The NMDAR is highly complex and adaptable. The excitatory neurotransmitter glutamate activates the NMDAR and requires glycine as a co-agonist. It is permeable to Ca2+ and utilizes extracellular Mg2+ as a voltage-sensitive block [26]. The receptor is composed of a combination of the subunits NMDAR1 (NR1), NMDAR2 (NR2), and NMDAR3 (NR3). These subunits are further categorized into different isoforms. For instance, NR2 can be further divided into NR2A, NR2B, NR2C, and NR2D. The receptors display a high level of versatility by modifying the expression of different subunits to accommodate certain functions. In general, the NR1 subunit binds the co-agonist glycine while the NR2 subunit binds glutamate [27].

A relevant example of the versatility of NMDAR is that certain subunits are strongly associated with the antidepressant effects of NMDAR blockade. A study investigated the use of a selective NR2B subunit antagonist called CP101,606. This study found that CP101,606 caused a significant reduction in MDD symptoms without causing dissociative adverse effects [28]. Similarly, another study found that subunit-specific blockades of either NR2B or NR2A had anti-depressant effects when inhibited independently; however, both subunits must be blocked simultaneously to elicit psychotic symptoms [29]. What this tells us is that activation of different subunits can impact psychiatric illness uniquely.

**Figure 1:**
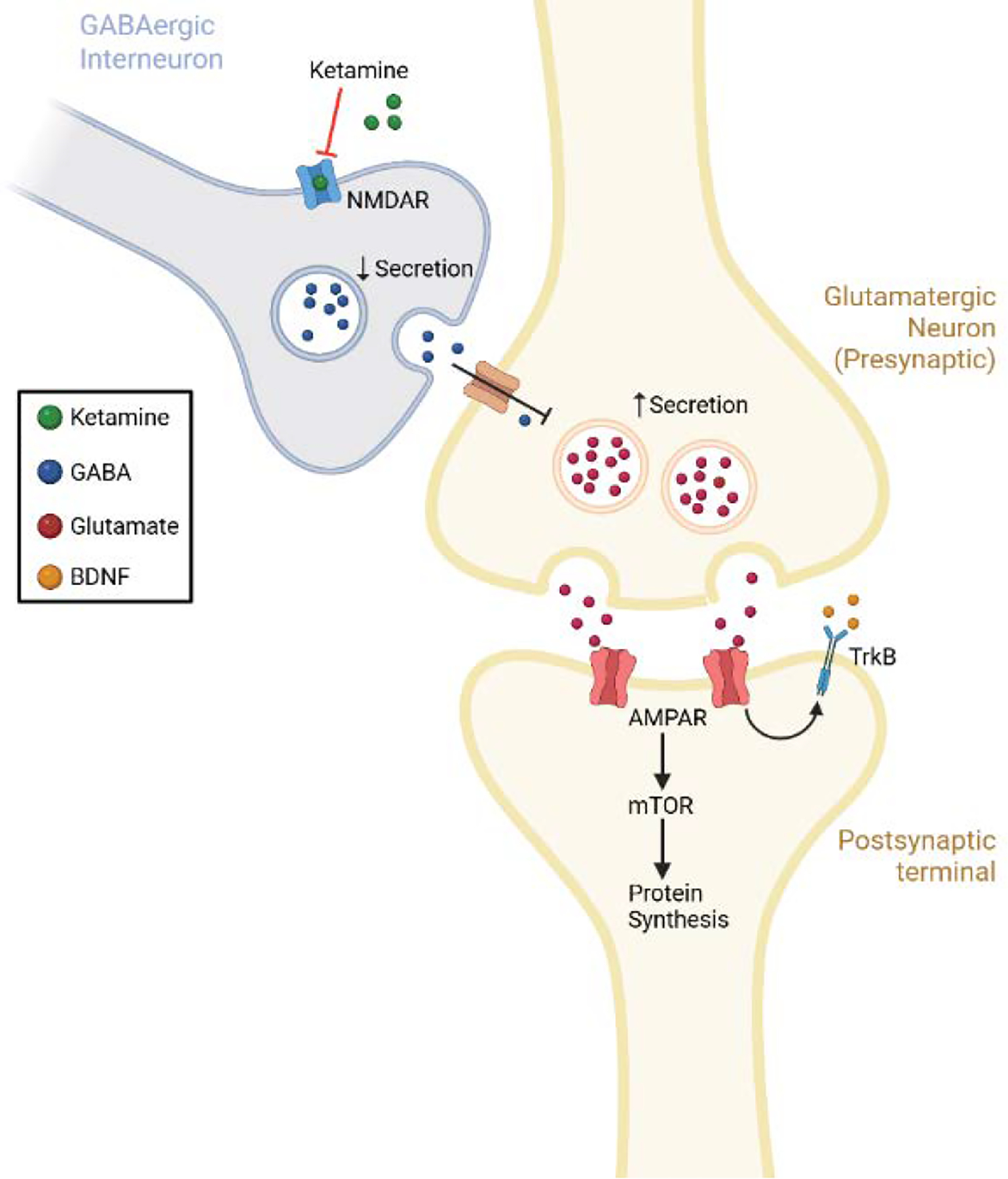
Ketamine causes disinhibition of glutamatergic neurons through its inhibition of GABA interneurons that results in increased BDNF and mTOR activity leading to protein synthesis and synaptogenesis.

The two key molecules in low-dose ketamines attenuation of MDD and PTSD are brain derived neurotrophic factor (BDNF) and mammalian target of rapamycin (mTOR). The proposed mechanism is that ketamine inhibits NMDAR expressed on the surface of gamma-aminobutyric acid (GABA) secreting interneurons, causing a decrease in GABA secretion. This decrease in GABA secretion results in disinhibition of presynaptic glutamate secreting neurons, ultimately leading to an increase in glutamate secretion (Figure 1). Glutamate then activates α-amino-3-hydroxy-5-methyl-4-isoxazolepropionic acid (AMPA) receptors on the postsynaptic neuron. Increased glutamate activation of AMPA receptors triggers a cascade thought to increase BDNF via its receptor tropomyosin tyrosine kinase B (TrkB). This enhances mTOR pathway signaling and subsequently increases synaptic protein synthesis (Figure 1). This leads to synaptogenesis and synaptic spine maturation [21], both of which contribute to brain plasticity.

## Plasticity and Metaplasticity of Ketamine

Dysfunction of metaplasticity and plasticity is involved in the pathophysiology of both MDD [30] and PTSD [31]. Furthermore, ketamine can regulate plasticity through its actions as an NMDAR antagonist. It is thus important to investigate ketamines ability to modulate PTSD in the context of brain plasticity and metaplasticity.

NMDAR is involved in brain plasticity, which occurs in the form of long-term potentiation (LTP) or long-term depression (LTD). Brain plasticity is thought to be involved in cognitive processing and encoding of long-term memories. Metaplasticity is another pathway modulated by NMDAR and is the process by which synapses are either weakened or strengthened to better suit a person’s memory and cognitive requirements. Essentially, metaplasticity regulates plasticity [32] and the effects of ketamine on NMDAR allow it to modulate both plasticity and metaplasticity.

Ketamine can regulate plasticity through its inhibition of NMDAR and subsequent activation of BDNF and mTOR [33,34]. As discussed in a previous section, these proteins interact with GABAergic and glutamatergic neurons to effectively increase synaptic protein synthesis, synaptogenesis, and synapse maturation [21]. These are the processes that lead to plasticity. This means that ketamine’s effects on NDMAR, BDNF, and mTOR can result in changes of plasticity and metaplasticity which are both involved in the pathophysiology of PTSD.

An increase in plasticity is thought to play a vital role in the therapeutic effects of subanesthetic ketamine on PTSD [21] and MDD [35]. This is supported by the observation that BDNF is decreased in patients with MDD and increased with effective treatment of MDD [35]. There is also evidence suggesting decreased BDNF in military personnel suffering from PTSD [36]. Although subanesthetic doses provide therapeutic effects through these pathways, anesthetic doses do not.

The ability of ketamine to modulate brain plasticity is dependent on the dose used. Specifically, synaptogenesis via activation of BDNF and mTOR is observed with low-dose but not high-dose ketamine. Moghaddam et. al. [37] found that anesthetic doses of ketamine actually decreased extracellular levels of glutamate in the prefrontal cortex of mice. Meanwhile, subanesthetic doses caused significant increases in extracellular glutamate. Decrease in glutamate with anesthetic doses would decrease the activity of BDNF and mTOR. This could impair synaptogenesis and brain plasticity, theoretically worsening PTSD. This is one proposed mechanism for why anesthetic doses do not have mitigating effects on PTSD.

Zorumski et. al. [38] made an astute observation that at low doses of ketamine, only a fraction of NMDA receptors are inhibited, and the remaining receptors can still be activated. A review article concluded that NMDAR antagonists block LTP at high doses but promote LTP at low doses [39]. It’s possible that the activation of the unblocked receptors with low-dose ketamine is what promotes LTP, and effectively brain plasticity. This increased LTP might play a role in ketamine’s therapeutic effects on psychiatric illnesses. Conversely, high-dose ketamine completely blocks NMDAR which inhibits LTP, potentially having negative impacts on illnesses like PTSD by reducing brain plasticity.

In summary, anesthetic doses of ketamine result in an overall decrease in glutamate in certain regions of the brain which causes a decrease in plasticity and metaplasticity. Another mechanism by which anesthetic doses of ketamine reduce plasticity is via complete blockade of NMDAR causing a decrease in LTP. The decrease in plasticity is thought to worsen symptoms of PTSD. This is compared to subanesthetic doses which have been proven to do the opposite. Therefore, it’s logical to conclude that anesthetic doses of ketamine will not improve PTSD and may instead worsen PTSD through its modulation of brain plasticity.

## Psychomimetic Symptoms

To understand how ketamine-induced anesthesia affects patients with PTSD it is important to discuss the frequency of psychomimetic symptoms at varying doses. In general, anesthetic doses of ketamine cause greater psychomimetic symptoms than subanesthetic doses. This increase in psychomimetic symptoms (i.e., hallucinations, delusions, negative symptoms) can function as unconscious reminders of the initial trauma event that led to the development of PTSD.

Bowdle et. al. [40] suggests a linear relationship between the steady-state plasma concentration of ketamine and the severity of psychomimetic symptoms experienced in healthy human volunteers. They measured this using a visual analog rating scale (VAS) and hallucination rating scale (HRS). The VAS included scales for items like “my body parts seemed to change their shape or position” and “I had feelings of unreality” for a total of 13 different scales. They found that there was a linear increase in all VAS items as the steady-state plasma concentration of the drug increased. There was also a significant increase in HRS compared to saline control.

Researchers intentionally induced a coma in patients using anesthetic doses of ketamine for 5–7 days to assess chronic pain response. In this study, psychomimetic symptoms were common after waking from the coma, specifically, nightmares, dysphoria, issues with sleeping, and anxiety [41]. These symptoms resemble those experienced by patients suffering from PTSD. This is compared to the common side effects seen in low-dose ketamine, which include drowsiness, dizziness, poor coordination, blurred vision, and feeling strange or unreal. At almost negligible rates, psychomimetic side effects were also seen at low doses [42].

A study investigating the side effect profile of low-dose ketamine found a small but statistically significant increase in dissociative and psychotomimetic symptoms. The study used CADSS to measure dissociative symptoms and the brief psychiatric rating scale (BPRS+) to measure psychotomimetic symptoms. They considered the increase in dissociative symptoms to be mild and the increase in psychotomimetic symptoms to be even smaller [42].

Another study found a linear increase in response to low-dose ketamine in patients with MDD such that 0.4 mg/kg caused a greater reduction in symptoms than 0.1 mg/kg [43]. Since they share similar pathways, we might expect to see that increasing doses of ketamine may result in further improvement of PTSD as well. This could be true if the dose stays within the subanesthetic range. However, at higher doses, we know that the frequency of certain side effects like dissociation changes abruptly [44]. Also, there’s evidence to suggest that high-dose ketamine does not exhibit anti-depressant activity and is thus unlikely to improve PTSD symptoms [20].

Low doses cause analgesia with mild sedation, but once a threshold of 1–1.5 mg/kg is exceeded, dissociative symptoms have an abrupt onset [44]. It was also noted that the dissociative symptoms experienced are distinct from those commonly seen in classical sedation and anesthesia. This is important because although low-dose and high-dose ketamine may have similar side effects, the frequency of these side effects may change abruptly at anesthetic doses.

This increase in unwarranted psychological experiences could impact PTSD patients differently than low-dose use of the drug. The psychodysleptic symptoms that are common with anesthetic doses of ketamine resemble the symptoms experienced by PTSD patients. In fact, the Clinician-Administered Dissociative States Scale (CADSS), which is a scale commonly used to assess ketamine’s dissociative symptoms, was originally validated in patients with PTSD [45]. It could be postulated that anesthetic doses of ketamine could increase exposure and remembrance of distressing traumatic events through the increase in psychomimetic and dissociative experiences. Ultimately, this could trigger worsening symptoms of PTSD.

## Memory and Fear

Although the pathophysiology of PTSD is complex and remains largely unknown, it is sometimes referred to as a fear-related psychopathy resulting from fear memory system dysregulation [46]. Similarly, there are multiple theoretical mechanisms by which anesthetics, including ketamine, can modulate memory and fear. Discussing the interplay between PTSD and ketamine in the context of fear and memory is critical to understanding their effects on one another.

**Figure 2:**
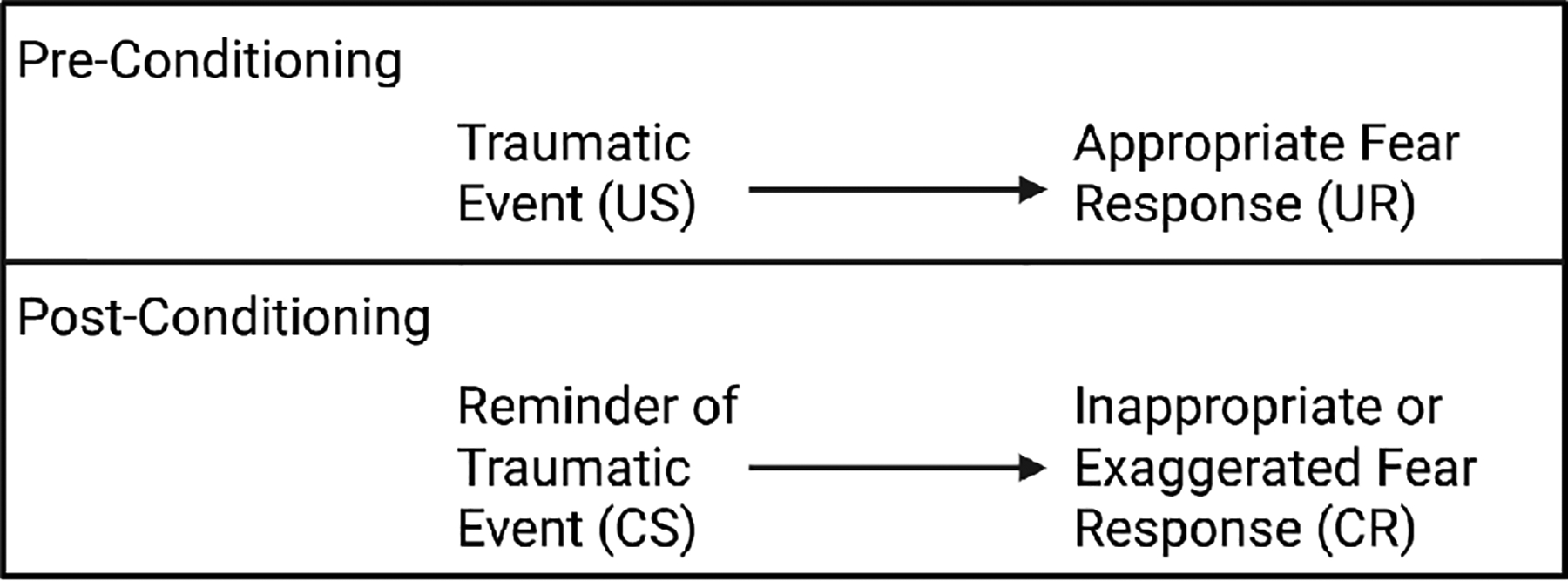
The initial traumatic event causes an appropriate fear response that becomes exaggerated with subsequent exposure to events that serve as reminders of the initial event. CR, conditioned response; CS, conditioned stimulus; UR, unconditioned response; US, unconditioned stimulus.

Some theories suggest the development of PTSD occurs after exposure to a traumatic event in which the fear response is exaggerated and persistent. Theoretically, this is because the trauma event (unconditioned stimuli) causes an appropriate fear response (unconditioned response), this subsequently develops into an inappropriate fear response (conditioned response) to events experienced in everyday life that are related to the initial trauma (conditioned stimuli) [47]. This can cause patients to have pathological fear responses to conscious or unconscious reminders of the initial traumatic event (Figure 2).

The conditioned fear response will continue until extinction memory is effectively learned. Extinction memory is when continued exposure of the conditioned stimuli in the absence of the unconditioned stimuli eventually diminishes or eliminates the conditioned response, or inappropriate fear in this scenario [48]. Factors that inhibit extinction memory formation could result in persistent inappropriate fear responses that can lead to the exacerbation of PTSD symptoms (Figure 2). One possible factor is the use of ketamine and other NMDA antagonists.

Ketamine has been shown to improve fear memory extinction at low-doses in rat models [49]. An increase in BDNF [50] and mTOR [51] activity are thought to be involved in this acceleration of extinction. This suggests that low-dose ketamine could improve PTSD symptoms by increasing the rate of fear memory extinction by enhancing BDNF and mTOR. However, the effects of high-dose ketamine and extinction are less known. At a molecular level, high doses might decrease BDNF and mTOR activity due to decreased glutamate [37] so we might expect that this would prolong fear memory extinction. More studies comparing fear memory extinction at high and low doses of ketamine are needed before any conclusion can be made.

One study found that NMDA antagonist MK-801 prolonged fear memory extinction while partial agonists accelerated fear memory extinction [6]. Being said, low-dose ketamine leaves unblocked receptors capable of being activated [39], which may function similarly to a partial agonist and result in accelerated extinction. Conversely, high-dose ketamine causes complete blockade and might prolong extinction just as MK-801 did in the experiment. Additionally, a study assessing the effects of the NMDA antagonist MK-801 in rats found that memory extinction was suppressed more with higher doses of the antagonist [52]. This is evidence that the antagonism of NMDAR can affect extinction differently depending on the dose. Unlike subanesthetic doses of ketamine, anesthetic doses could delay extinction and worsen PTSD in the context of fear memory dysregulation.

The various subunits of the NMDAR serve unique roles in memory formation and maintenance. Can et. Al. [53] found that the NR2A and NR2B subunits in the hippocampus of mice affected fear memory differently. Specifically, selective NR2B antagonists led to impairment of acquisition and long-term storage of trace fear memory but not contextual fear memory, while NR2A antagonists impaired both trace and contextual fear memory. This means that specific NMDAR subunits can distinctly modulate fear memories.

To summarize, the effects of NMDAR antagonists, such as ketamine, have complex interactions with fear memory systems that are not fully understood. The acceleration of fear extinction by low-dose ketamine could reduce the maladaptive fear response and improve PTSD. It is thought to do this via activation of BDNF and mTOR pathways. Since high-dose ketamine reduces the activity of these pathways we could infer that anesthetic doses would prolong extinction and worsen PTSD. There is also some evidence suggesting that higher doses of NDMA antagonists do indeed impair extinction in rat models. More research directly measuring the effects of varying doses of ketamine on extinction and the implications this has on PTSD is required before a conclusion can be drawn with certainty.

## Conclusion and Future Direction

In this article a critical discussion is presented on the effects of anesthetic doses of ketamine on patients who suffer from PTSD. Based on the limited information in the literature, anesthetic doses of ketamine could potentially worsen PTSD, while subanesthetic doses can improve PTSD. However, it is difficult to definitively answer this question because there is currently no research directly measuring this relationship. Certain assumptions can be made using current evidence-based research and we approached this through multiple lenses. One being the ability of ketamine to modulate brain plasticity through changes in glutamate, BDNF, and mTOR. In this regard, the increased plasticity with low-dose ketamine is thought to mitigate PTSD. However, at high-doses elicit opposite effects and there is a decrease in plasticity due to reduced glutamate release, likely worsening PTSD symptoms. The second approach was ketamine’s increase in psychomimetic symptoms at anesthetic doses. Studies show that psychomimetic symptoms have an abrupt increase in frequency at higher doses. These enhanced psychomimetic symptoms can serve as unconscious reminders of the initial trauma, triggering symptoms of PTSD. Lastly, the ability of ketamine to influence fear memory systems is less understood but there may be prolongation of fear memory extinction with higher doses of ketamine. This would lead to delayed resolution of PTSD. Thus, there are multiple ways by which ketamine could theoretically worsen PTSD; however, more careful and well-controlled studies directly measuring the effects of ketamine on PTSD are warranted before a definitive conclusion can be made.

There are currently no published research articles investigating the effect of anesthetic doses of ketamine on PTSD. The current emphasis in research has been on subanesthetic doses of ketamine and its potential as rapid treatment for psychiatric disorders like MDD and PTSD. One reason for this is that ketamine-induced general anesthesia is associated with increased morbidity and mortality making it difficult to conduct such a study [56]. To avoid unnecessary harm, studies could be conducted that analyze patients undergoing planned surgeries where ketamine will be used as the anesthetic. Another barrier is that patients under ketamine-induced anesthesia are unconscious and incapable of providing real-time subjective information. Although there is no way to avoid this, real-time subject information is not necessary to investigate the relationship between ketamine and PTSD. Questionaries after conscious is regained or measurements of molecules like BDNF and glutamate could provide sufficient information to further understand the effects of ketamine. There is also a lack of studies in non-human models. Studies designed using animal models such as rats could help the understanding of the molecular impacts of anesthetic doses of ketamine and the impact of ketamine on fear memory extinction. Although barriers exist, there are still many valid approaches to safely and effectively measuring the impact of anesthetic doses of ketamine on PTSD.

Understanding the effects of high dose ketamine on PTSD patients is important for anesthesiologists to successfully implement ketamine as an anesthetic agent. As discussed, there are many benefits to using ketamine as an anesthetic and advancing our knowledge and understanding of its effects on certain patient populations will allow us to use it more often. As an example, it is common practice that bolus doses of ketamine should be avoided in schizophrenia due to its worsening effects on this disease [6]. Similarly, if PTSD was found to be exacerbated by anesthetic doses of ketamine it would be wise to change guidelines to accommodate this finding. For instance, there might be a recommendation against the use of ketamine in patients with PTSD, or possibly even a contraindication. This would encourage anesthesiologists to obtain a thorough psychiatric history to appropriately provide anesthesia to the patient. Also, the psychomimetic effects of ketamine could be attenuated by pretreatment with midazolam [54]. This could allow for PTSD patients to safely receive anesthetic doses of ketamine. Gaining a better understanding of the PTSD and ketamine relationship could lead to improved patient outcomes and an overall decrease in morbidity and mortality in patients with PTSD requiring surgical intervention.

## Key Points

- Ketamine is a desirable drug to use during anesthesia due to its relatively mild side effect profile and its analgesic, amnesic, and anesthetic properties.
- Ketamine at low-doses has shown to be therapeutic in illnesses like PTSD and MDD.
- Subanesthetic ketamine’s therapeutic effects are likely due to its modulation of brain plasticity through increases in glutamate, BDNF, and mTOR.
- Anesthetic doses of ketamine cause decreases in glutamate and subsequently BDNF and mTOR which may result in worsening PTSD symptoms.
- Other mechanisms by which anesthetic doses of ketamine could worsen PTSD is through its increase in psychomimetic symptoms and prolongation of fear memory extinction.
- Overall, high-dose ketamine most likely does not improve symptoms of PTSD and could instead worsen them.
- More studies directly measuring the effects of anesthetic ketamine are necessary to definitively conclude whether it has exacerbating or mitigating effects on PTSD.
